# Supporting Public Health Research Capacity, Quality, and Productivity in a Diverse Region

**DOI:** 10.2196/39154

**Published:** 2023-07-31

**Authors:** Rana AlHamawi, Randa K Saad, Hanan F Abdul Rahim, Khwaja Mir Islam Saeed, Abdullatif Husseini, Yousef Khader, Mohannad Al Nsour

**Affiliations:** 1 Global Health Development/Eastern Mediterranean Public Health Network Amman Jordan; 2 Qatar University Health College of Health Sciences Qatar University Doha Qatar; 3 Afghanistan Field Epidemiology Training Program Afghanistan National Public Health Institute kabul Afghanistan; 4 Institute of Community and Public Health Birzeit University Ramallah Occupied Palestinian Territory; 5 Public Health Faculty of Medicine Jordan University of Science and Technology Irbid Jordan

**Keywords:** health research, Public health, Eastern Mediterranean region, Research capacity, Research Quality

## Abstract

Public health research plays a critical role in strengthening health systems and improving their performance and impact. However, scholarly production in public health coming from the Eastern Mediterranean Region (EMR) remains well below the world average and lacks a tangible growth trend over time. During the seventh Eastern Mediterranean Public Health Network Regional Conference, a roundtable session brought together a panel of public health experts representing Global Health Development/Eastern Mediterranean Public Health Network affiliates, universities or academia, and research institutions from the region, where they shared insights on the current situation of public health research; challenges and barriers to research facing the different countries in the EMR and the region in general; and how research agendas, productivity, and quality can be supported through strengthening research capacity in the region. Although the region is diverse in terms of health system capacity and socioeconomic development, several common challenges were identified, including a lack of strategic prioritization to guide health research, insufficient funding, ineffective transfer of knowledge to policy and practice, limited availability of research facilities, and limited national and international research collaboration. Occupied countries and countries in a state of conflict, such as Palestine, face additional barriers, such as personal and social security, lack of control of borders and natural resources, travel and movement restrictions, and confidentiality challenges because of the continuing war conditions and occupation. However, there have been success stories in the EMR regarding research publications and their positive and effective impact on policy and decision-makers. To improve research resilience and public health care in the region, a collaborative approach involving institutions, policymakers, and relevant stakeholders is critical.

## Introduction

Public health research plays a critical role in strengthening health systems, improving their performance and public health impact, and adding value to society, as it provides critical information regarding disease trends, risk factors, outcomes of treatments or health interventions, patterns of care, and health care use and costs [1]. The importance of building research capacity in low-and middle-income countries (LMICs) has been recognized for well over 2 decades. The 1990 Commission on Health Research for Development reported that strengthening research capacity in LMICs is “one of the most powerful, cost-effective and sustainable means of advancing health and development” [2]. Indeed, locally led health research is important in overcoming global health barriers and challenges in LMICs [3].

Researchers usually initiate research studies with the best of intentions. However, the research process may face several challenges that hinder attempts for achieving successful results, especially in a region as diverse and facing as many political and financial challenges as the Eastern Mediterranean Region (EMR), which includes 22 countries as defined by the World Health Organization [4]. Therefore, research efforts and capacities in the EMR must be comprehensively reviewed within a national, regional, and global context.

This viewpoint aims to share insights from a roundtable discussion on the challenges and barriers facing public health research in the EMR, in particular the LMICs, and share views on what can be done to strengthen research capacity and improve research quality and productivity in the EMR.

## Roundtable Description

### Overview

A roundtable session was held on November 18, 2021, as part of the Seventh Eastern Mediterranean Public Health Network (EMPHNET) Regional Conference. The conference was attended by many experts from ministries of health, academic institutions, and public health institutes who represent most of the EMR. The roundtable brought together a panel of public health experts representing Global Health Development (GHD)|EMPHNET affiliates, universities or academia, and research institutions at regional and global levels. It provided a space for sharing insights on the challenges and barriers to research in the EMR and how research agenda can be supported by strengthening research capacity and increasing research productivity and quality in the region. The roundtable included oral presentations and an interactive discussion of questions and comments from participants. The following topics were presented to address the roundtable objectives: barriers and challenges to public health research in the EMR and strategies for improvement; investing in public health research and the COVID-19 response; a perspective from Qatar; public health research under occupation; an example from Palestine; and public health research in the EMR and the contribution of GHD|EMPHNET.

### Public Health Research in the EMR

Public health research and scientific production coming from the Middle East remains well below the world average and has not increased significantly over time [5]. The global share of EMR health research publications is much smaller than its global share of population or wealth. Between the years 2004 and 2013, the EMR contributed to only 0.85%-2.36% of total PubMed publications in health, and only 5 countries in the region accounted for 80% of the total publications: the Islamic Republic of Iran (39%), Egypt (14%), Saudi Arabia (11%), Tunisia (8%), and Pakistan (8%) [6]. Additionally, even though the population of the EMR has reached nearly 745 million people [4], only 2.35 articles were published per 100,000 population per year during 2003 to 2013 [6]. The low number of publications and lack of a comprehensive contribution from all countries in the EMR can be attributed to the several challenges faced in the region.

### Barriers and Challenges to Public Health Research in the EMR and Strategies for Improvement

Table 1 illustrates some of the challenges and barriers affecting the quality, capacity, and productivity of research in the EMR, including strategies for mitigating these barriers and improving overall research in the region.

**Table 1:** Barriers facing research in the Eastern Mediterranean Region and strategies for improvement.

Barriers	Possible strategies for improvement
Lack of an identified research agenda based on emerging priorities [7-9]	Set national research priorities and raise awareness about the importance of research for evidence-based decisions and policies [7,10]
Limited national and regional strategies for health research, and a lack of national policies and regulations that govern the conduct of health research [7,9,11]	Adopt national plans, strategies, and policies for effective health research [10] Adopt a regional strategy between supranational organizations and groupings to steer improvements in health research and encourage effective research stewardship in the Eastern Mediterranean Region [11].
Ineffective knowledge transfer to policy and practice [7,9,11]	Build capacities of policymakers to demand information for decision-making and carry out informed decision-making [12] Develop knowledge translation platforms to support the dissemination of evidence-based data and facilitate the dialogue between researchers, policymakers, and relevant stakeholders [13]
Limited availability of research facilities, equipment, and resources [9]	Foster an appropriate and effective research environment by advocating for national investment in public health infrastructure, such as research infrastructure and resources (libraries, scientific literature, communication facilities) [11,14], and maintain it by encouraging regional collaboration of countries with different resource levels.
Limited collaboration between research institutions and other national, regional, and international research institutions [9]	Develop and share a database of researchers and their expertise [15] Build capacities in grant writing and ensure that researchers from the region engage in international platforms, including conferences and meetings, to advocate for the Eastern Mediterranean Region’s issues [16]
Insufficient human resource capacity regarding research skills and competencies; lack of overall human resources [9,17,18]	Building capacities in research should be tied to the educational system, especially higher education. Academic research centers or institutes should focus on capacity building, improving scientific writing or publishing, health research proposal writing or funding, and quantitative and qualitative research methods. Continuous learning should be adopted as a strategy to build research capacities [19] Organize training courses for researchers and editors, such as “train the trainers” courses [20]
Limited national and regional research funding and ongoing sustainability of funding [8,11]	Establish a research finance system using innovative revenue generation [21] Advocate for funding through shared causes and engaging with the media [22]
Inadequate mentorship [9]	Provide recognition and long-term funded positions to mentors [23] When mentoring is locally unavailable, peer mentorship or institutional exchange or partnerships can be implemented [24]
Difficulties in retaining qualified researchers, research assistants, and associates (brain drain). As well as a lack of motivation and incentives to encourage researchers to conduct and engage in public health research [25]	To discourage brain drain, countries should ensure an entire ecosystem exists that supports and strengthens research, including making available research support (funding), human resource support (qualified research assistants), opportunities for dissemination and networking, physical infrastructure, and research compliance mechanisms [26] Provide higher salaries and link research production and publication to academic promotion [27]

Fostering and promoting an efficient environment appropriate for health research is required for planning, designing, and implementing research, disseminating evidence-based data, and translating the findings into evidence-informed policies and decisions [7]. One of the challenges facing the advancement of research in most countries in the EMR is that health and health research are not seen as a priority in terms of funding by most governments, the private sector, or the third sector (organizations). Investments in research systems are low in comparison to health in general [11]. Evidence has shown that research and development in the EMR is among the lowest in the world with an average of 0.3% of the region’s gross domestic product, which is well below world leaders such as Japan (2.8%) and the United Kingdom (1.8%) [8]. Research institutions conducting quantitative or qualitative research are receiving limited national, regional, and international funds [11]. The limitations in national funding can be due to national governments and policymakers allocating limited budgets for health research [2,8,11], policymakers being unaware of the value and importance of health research in improving public health and health systems, lack of engagement of policymakers in health research, and lack of a national health strategy and policies that define research priorities and guide health research in the region [7,9,11]. Indeed, previous research has shown that only 3 out of 10 EMR countries had reported setting national research priorities and only 2 countries had a national health research policy in place [5,8]. Additionally, it has been shown that only 29.3% of research institutions in the EMR reported having policymakers as part of their advisory board [11].

The low rate of investment of governments in health research is becoming apparent in the productivity of health research in the EMR. Previous research has shown that the research productivity of the EMR is very low next to international comparators (Europe, the Americas, and the Western Pacific). The EMR is only comparable to Africa and Southeast Asia. However, it is still behind the latter [28,29]. Additionally, research productivity in the EMR is not evenly distributed among countries, with Egypt and Pakistan contributing around 62% of the articles in the Index Medicus, while Afghanistan, Djibouti, and Somalia have not contributed any articles to the Index Medicus [30]. Evidence has shown that the publishing of biomedical and health research is directly linked to the state of socioeconomic development and political situation of the country [31,32]. The 3 countries that have contributed the least are among the least developed in the world. Somalia specifically has been experiencing a protracted emergency (a civil war) since the 1990s. The civil war, accompanied by famine and population displacement, has severely affected Somalia’s health system. In 2010, the transition to recovery of the health system organization, regulation, and workforce development began, with the institution of a transitional federal government. This transition created opportunities to initiate the pursuit of universal health coverage. During this phase, around 25 academic institutions with undergraduate medical or health courses and a few master’s courses were operationalized, in addition to reviving the collaboration between Swedish universities that was offset by the civil war [33,34]. There have been some additional efforts to accelerate and prioritize research in Somalia; for example, in 2022, the Somali National Institute of Health (NIH) and Federal Ministry of Health organized Somalia’s first NIH Health Research Conference in collaboration with multiple actors such as the World Health Organization, the Alliance for Health Policy and Systems Research, the Public Health Agency of Sweden, the African Field Epidemiology Network, Somali universities, the Somali-Swedish Research Cooperation Initiative, and the Somali Swedish Researchers’ Association [35]. The conference placed research at the forefront to accelerate progress toward universal health coverage [35]. The *Somali Health Action Journal*, established in collaboration between Somalia and Swedish universities, was showcased during the conference. The *Somali Health Action Journal* offers an open-access dissemination platform for addressing Somalia’s health challenges [35,36]. Despite all these efforts, Somalia’s national health system remains fragile, and national public health research is an essential component toward health system resilience.

Another barrier to a resilient public health system in the EMR is that research agendas in LMICs are set by international funders rather than local research institutions. Furthermore, the research implemented in LMICs is mainly led by researchers from high-income countries (HICs), with little contribution from LMIC researchers [9]. The small contribution from LMICs in conducting the research can create what is known as “parachute research,” where research is collected in LMICs but further work is carried out in HIC research institutions. This can severely diminish research capacity development [37] within the EMR and implement priorities that match the funder’s priorities than the actual health needs of the region [17].

The research in the EMR experience from lack of knowledge dissemination and transfer to policy [9], lack of national policies and regulations that govern the conduct of research [7,9,11], brain drain of researchers to HICs [25], lack of mentorship [9], and insufficient human resource competencies and skills [9,17]. For research to be effective in the EMR, a systems approach to capacity building should be adopted [38]. This approach addresses the national research system on 3 levels: macro, institutional, and individual. For health research to contribute to the reduction of health inequalities, research must be conducted on the basis of a research system that has well-defined goals and priorities [39]. To develop such a resilient system, countries must invest in capacity building at the macro level, where leadership and management skills are fostered [40]. At the institutional level, countries must focus on fostering an appropriate and effective research environment for their researchers. For example, having adequate and appropriate research infrastructure and resources (scientific literature, communication facilities) and maintaining the continuity of funding [14]. The individual level focuses on building technical competence (in data analysis and protocol development) but also builds the individual’s capacities in other aspects of research such as priority setting, networking and leadership, disseminating and translating knowledge, and partnership development. This level is not only targeted at researchers but must include other stakeholders, such as decision-makers, health workers, research managers, and community members [14].

The unstable political leadership in the EMR is another significant barrier for public health research. In countries with unstable political leadership, research funding and priorities are inconsistent and unpredictable, making it difficult for researchers to plan and conduct their work effectively. Additionally, political instability can create an environment of insecurity and uncertainty, which can discourage international collaboration and investment in research. Political instability can also lead to brain drain, as researchers may leave the country in search of more stable and supportive research environments.

### Public Health Research Under Occupation: An Example From Palestine

It is evident that colonial structures where indigenous people live foster material and social inequalities, which lead to health disparities that persist over several generations. Diminished life expectancy, a disproportionate burden of communicable and noncommunicable diseases, social violence, and addiction have been linked to colonial structures [11]. “Research has increasingly established that poor health outcomes in Indigenous peoples, and the health disparities realized by Indigenous peoples in almost all sectors of life as compared with their nonindigenous counterparts, stem from or are related to colonial disruptions and ongoing erosion of human rights” [41]. Therefore, encouraging research in such countries is critical for the advancement of the health of indigenous people. However, public health research is severely affected in occupied countries such as Palestine, where fragmentation of communities, land, and the health system and dependence on international aid are prevalent.

Despite the benefits of research in conflict zones, researchers are faced with challenges at every step of the research process, from conducting fieldwork and disseminating research findings to the repatriation of researchers [40]. Researchers conducting research in occupied countries such as Palestine are faced with the abovementioned barriers and challenges. However, due to the continuing war conditions and the Israeli military occupation in Palestine [42], they are also faced with personal and social security and confidentiality challenges. For example, safety is a significant issue facing both researchers and communities in conflict zones. Researchers should sometimes avoid the use of participatory methods such as the gathering of many people in one place, as they represent high-risk strategies in areas subject to military aggression [14]. Researchers must also be reflective on where and how they conduct research, what they talk about, and who they talk to to avoid jeopardizing communities’ safety. Researchers must be thoroughly trained on how to conduct research in conflict zones and should develop adequate skills and competencies that allow them to accurately assess the political situation. They should be able to identify which topics are too sensitive to talk about and can therefore put lives in the communities at risk [14,43]. Additional obstacles related to conducting research in occupied countries include a lack of control of borders and natural resources and travel and movement restrictions. This may affect sample transportation to other countries and purchasing equipment needed for research, causing delays and affecting the research timelines and outcomes. Finally, the dependence on international aid to fund health research, which may have its own research agenda, may not be compatible with occupied countries’ research needs and agendas.

Communities living in conflict zones might revert to a strategy of silence, where they keep a low profile and mind their own business to protect themselves from militarized violence, including ethnic cleansing and demonstrative killings. Reverting to silence might also be a coping mechanism used by traumatized individuals. Therefore, researchers must be aware of the sensitivity of the collected information and the fears of the communities and respect the boundaries of the individuals. Researchers might be faced with challenges when it comes to disseminating such sensitive information for the greater good of these communities without risking the welfare of research subjects [14].

Researchers must follow the “do no harm” strategy in conflict zones to reduce the negative impact of research. This strategy requires the selection of well-trained and mature researchers that are aware of ethical dilemmas. It also involves a balance between insider and outsider researchers with relevant ethnic backgrounds and language, cultural sensitivity, and religious skills. Researchers are required to blend in with their surroundings, keep a low profile, prepare methodological contingency plans, frequently monitor the political situation, analyze risk, obtain informed consent, and maintain confidentiality [14,43].

### Benefits of Investing in Public Health Research: A Perspective From Qatar

Despite the challenges that face the EMR, there have been some success stories. Qatar’s response to the COVID-19 pandemic has been effective due to a number of factors, including the use of high-quality epidemiological and clinical data to support the national response efforts. In the first wave of COVID-19, the number of COVID-19 cases in Qatar was especially high among craft and manual workers living in dormitory-like conditions. However, case fatality has been among the lowest globally [7]. The success of the national COVID-19 response can be attributed to the interplay of a number of factors, including (1) linking science to policy through a multi-stakeholder platform as part of an effective governance mechanism, where scientific evidence was used to direct appropriate public health and health care measures against the pandemic; (2) providing rapid and flexible research funding (the Qatar National Research Fund established a rapid response call, offering researchers grants of up to US $27,460 each for impactful 3-month projects related to COVID-19, and Qatar University offered an emergency response grant of up to US $39,000 for each COVID-19–related research project); (3) readying infrastructure for infectious disease research ahead of the pandemic, including the biosafety level 3 laboratory at Qatar University; and (4) ensuring the availability of centralized and complete data, where the national SARS-CoV-2 databases are integrated using a digital health information platform [44]. Because of these factors, scientists from Qatar were able to contribute to international scholarship on the unfolding pandemic through a number of high-profile publications. It was seen that gains made in COVID-19 research in terms of data access were linked to policy, and collaboration should be sustained and implemented in other research areas.

### Contribution of GHD|EMPHNET to Public Health Research

Despite the many challenges and barriers that face the EMR, GHD|EMPHNET was able to contribute to the improvement of the region’s public health resilience and strengthen public health researchers’ capacities. GHD|EMPHNET supports researchers through a hybrid model, where face-to-face and preconference workshops, Zoom, and Microsoft Team meetings are carried out whenever possible. For example, GHD|EMPHNET’s knowledge exchange network creates a space for field epidemiology training program residents and graduates to share and exchange their ideas, discuss their work, learn from one another, and improve their skills through continuous collaboration. It also provides technical support to researchers. It has formed a research group targeting priority areas that involves a core team of lead researchers and more than 40 coresearchers. It has also published its own e-book and its fair share of case studies and papers. GHD|EMPHNET launched its own webinar initiative, the EMPHNET WEBi series, that serves as a web-based dialogue opportunity to bring together a wide range of audiences. Its objectives are to enhance relationship and coordination between countries, stakeholders, and partners; maximize the use of available measures to enhance and develop public health expertise, capacity, and community awareness; and to disseminate information and knowledge applicable to the public health and EMR priorities and needs.

### Conclusions

There are many challenges and barriers facing public health research capacity, quality, and productivity in the EMR. The identified challenges in the EMR are a lack of national health strategy and policies that define research priorities and guide health research, lack of funding, lack of effective knowledge transfer to policy and practice, limited availability of research facilities and resources, limited national and international research collaboration, inadequate competencies of human resources, brain drain, and inadequate mentorship. Researchers conducting research in conflict contexts are faced with more challenges compared to many other settings, such as social security and confidentiality barriers. However, there have been success stories in the EMR regarding research publication and its positive and effective impact on policy- and decision-makers. To improve research resilience and public health care in the region, a collaborative approach involving institutions, policymakers, and relevant stakeholders is critical.

### Recommendations and Key Areas for Improvement

- Public health research capacity and competencies can be improved through collaborative work with local research teams. Collaboration with research team members and coauthors from credible international universities leads to improvements in research capacities and strengthens research in the region.
- In a conflict country, efforts to contribute to public health research in contexts of violence and political and economic oppression are of priority to the region, are highly valued by the global research community, and may merit the granting of research funds.
- Research in conflict areas needs to investigate the needs of people living in such areas and consequently the appropriate responses to their needs. Therefore, a framework should be developed to assist researchers committed to ethical decision-making.
- Official guidelines on data sharing should be developed, should be clear and consistent, and should balance between making data available and accessible to researchers and safeguarding privacy. A centralized mechanism such as a secretariat or a commission should monitor and facilitate data sharing among various stakeholders for efficient and fair use of data for the health of the community.
- Universities must make research skills and competencies part of the curriculum for undergraduate students. Efforts should be made to mentor undergraduate students and develop their research competencies. Mentors should be given incentives to encourage mentorship. Furthermore, universities should develop better archival databases and consequently provide better access to up-to-date data for their students.
- Research institutions must focus on developing a research capacity educational program such as “train the trainer,” where institutions adopting such a program must be flexible and willing to revise the plan if faced with barriers and challenges.
- Knowledge transfer frameworks and programs should be developed and implemented for collaborative knowledge transfer between researchers, policymakers, and other relevant stakeholders to facilitate the linkage of science to policy.

## Abbreviations

EMR: Eastern Mediterranean Region
GHD|EMPHNET: Global Health Development|Eastern Mediterranean Public Health Network
HIC: high-income country
LMIC: low-and middle-income country
NIH: National Institutes of Health
